# News and Miscellany

**Published:** 1900-06

**Authors:** 


### News and Miscellany.

---

**Dr. O. H. Radkey,** one of the recent graduates of the Medical Department of the University of Texas, has located at Austin.

---

**Change of Address.**—Dr. J. Landegren from Shafter, Texas, to Marfa, Texas. Dr. J. R. McCown from Hallettsville, to Kinkler, Texas.

---

**The name** of the *Alabama Medical and Surgical Age* has been changed to the *Alabama Medical Journal.* Dr. LeGrand got tired writing such a long name.

---

**Dr. Preston B. Scott,** of Louisville, who was medical director of the Confederate army in Mississippi, was, at the recent Confederate reunion, elected president of the Association of Confederate Surgeons.

---

**"When** will doctors understand that collections deferred maketh the patient dishonest?" —*Lydston.*

When will subscribers understand that payment deferred maketh ye editor tired?

---

**The** JOURNAL regrets to announce the death of Dr. J. P. Coffey, of Plano, Texas, at his home, May 26 ult. Dr. Coffey was the leading physician of his section of country, and was never a day without the "Red Back" from the beginning.

---

**End of Vol. 15.**—With this number the *Journal* completes its fifteenth year. We take occasion to extend our renewed thanks to the "faithful." We met many at Waco who said that they have every number of the "Red Back" that has ever been issued, and expect to take it as long as they live.

**Dr. Daniel's Book**—"Recollections of a Rebel Surgeon"—will be clubbed with the JOURNAL as an inducement to new subscribers, to begin with next number—July—which begins Vol. 16. The JOURNAL, 1 year, $1.00; the book, postpaid, $1.10, $2.10. Will send a limited number—both—for $1.60, cash with order.

---

**W. B. Saunders,** the well known medical book publisher of Philadelphia, announces that he has associated with him, under the firm name of W. B. Saunders & Co., Mr. F. L. Hopkins, manager of the subscription department, and Mr. T. F. Dagney, manager of the publication department. These gentlemen have been connected with the establishment almost from its inception, and to their efficiency Mr. Saunders attributes much of the success that has attended his efforts.

---

**The "Duke of Wellington."**—Sharp & Dohme have established a Southern branch of their business in New Orleans. The hosts of friends of Mr. J. A. Wellington, so long their representative in Texas, will be glad to know that he has been placed at the head of it. The "Duke" is, deservedly, one of the most popular traveling men that has ever been in Texas, and while we will miss his semi-occasional visits and his cordial, genial greeting, we extend him our congratulations on so well deserved a promotion.

---

**Love's Medical Mirror** (St. Louis), offers $500 for the best paper on consumption and $500 more for the next best three papers. See advertisement. Never since my day has there been so much interest in the subject of consumption. A crusade for its extermination is being inaugurated, and in all parts of the world the sanitary hosts are being mustered. The *Mirror's* enterprise is most commendable, and will stimulate research and renewed interest in the treatment of consumption. Send 10 cents for the June number of *Love's Medical Mirror*, which contains full particulars.

---

**Local Anaesthesia.**—For the ear, etc.

℞ Cocaine muriate . . . . . . . . . . . . . . parts 10.
Dilute alcohol . . . . . . . . . . . . . . . . parts 50.
Aniline oil . . . . . . . . . . . . . . . . . . parts 50.

M. Ft. solutio.

This solution is suitable for operations on the tympanic membrane, on granulations, or for the removal of the ossicles. If the membranes are dense increase to absolute alcohol and the amount of the oil and use a larger amount of the solution and apply longer.—*London Lancet.*

---

**Dr. Paschal's** paper in this issue is a most remarkable one, or rather it is a chronicle of a most extraordinary experience in lithotomy. It is as creditable to the doctor as it is unique in the history of stone. In forty-three lithotomies by the perineal method—all ages—from two and a half years to eighty,—operations done without antiseptic measures—on poor Mexicans, and often on an undressed sheepskin for a bed, he lost but two patients; both old men. Every surgeon should read this paper and preserve it. It should be extensively copied, and the record should be preserved in the text-books. Can anyone make a better showing? Illustrations of stones are natural size.

